# Construction and empirical study of China's snow sports evaluation Index system

**DOI:** 10.3389/fspor.2025.1619136

**Published:** 2025-11-07

**Authors:** Yi Nuo, Xue Han, Qiu Sen

**Affiliations:** 1 College of Education, Capital Normal University, Beijing, China; 2 College of Sports Science, Nantong University, Nantong, Jiangsu, China; 3 Winter Sport School, Hebei Institute of Physical Education, Shijiazhuang, China

**Keywords:** snow sports, evaluation system, interview method, grounded theory, Beijing Winter Olympics

## Abstract

**Introduction:**

This study aimed to construct a scientifically-grounded evaluation index system for snow sports development in China, addressing persistent challenges in mass participation, industry cultivation, and regional coordination despite competitive achievements. The research was motivated by the transformative impact of the 2022 Beijing Winter Olympics and the need for a locally adapted evaluation framework that maintains international comparability.

**Methods:**

A mixed-methods approach was employed, combining in-depth interviews with 43 experts analyzed via grounded theory to develop a three-level indicator system (3 primary, 13 secondary, and 54 tertiary indicators). A combined weighting method integrated objective data characteristics and normative design principles, applied to panel data from 2015–2019. Regression analysis was conducted to quantify the impact of Winter Olympics investment.

**Results:**

The overall development index increased 10.92-fold from 2015 to 2019 with mass participation showing the most rapid growth (12.71-fold) followed by competitive development (10.64-fold) and economic development (8.82-fold). Regression analysis confirmed that Winter Olympics investment had a significant positive impact (*β*= 0.1661, *p* < 0.1) while cost expenditure was a significant barrier (*β*= −0.0190 to −0.0398, *p* < 0.05).

**Discussion:**

Strategic Olympic investment catalyzed holistic development, particularly in mass participation, amplified by policy campaigns like “Engaging 300 Million People in Ice and Snow Sports.” The concentric circle model validated the interconnectedness of competitive, mass, and economic dimensions. However, high operational costs threaten long-term sustainability, necessitating policies to improve.

## 1 Introduction

The sustainable development of snow sports is intricately linked to natural environments, cultural traditions, and economic factors. Since China's debut at the 1980 Lake Placid Winter Olympics (1), the country's snow sports sector has undergone significant evolution over four decades. While notable competitive milestones have been achieved—exemplified by China's first Olympic gold medal in snow events at Beijing 2022—persistent challenges remain in mass participation, industry cultivation, and regional coordination (2). The successful bid for the 2022 Beijing Winter Olympics served as a transformative catalyst (3), prompting national strategic initiatives such as the “Engaging 300 Million People in Ice and Snow Sports” campaign (2018–2022) and the 14th Five-Year Sports Development Plan. These policies established a development model characterized by “competitive leadership–mass popularization–industrial synergy” (4), creating a practical foundation for sustainable growth in the post-Olympic era.

Globally, the assessment of mega-sporting event legacies provides valuable frameworks for evaluation. The International Olympic Committee's (IOC) Olympic Games Impact (OGI) study and subsequent legacy assessment methodologies emphasize the multidimensional economic, social, and environmental effects of such events (5). Comparative analyses of Vancouver 2010, Sochi 2014, and PyeongChang 2018 reveal a consistent structural pattern involving the interplay between “core competition,” “public participation,” and “industrial and environmental spillover” (6), which aligns with the conceptual approach adopted in this study. However, the Chinese context presents unique characteristics, including simultaneous phases of rapid infrastructure expansion and intensive policy support, set against a backdrop of considerable climatic and geographical diversity (7, 8). This necessitates a locally adapted evaluation framework that maintains international comparability while incorporating context-specific dimensions related to facilities and organizational structures.

Despite these advancements, a significant research gap persists in the systematic development of a comprehensive evaluation index system for snow sports in China (9). Existing studies often suffer from excessive subjectivity in indicator selection and weight distribution, lacking unified standards that would enable cross-validation and comparison of research outcomes (10, 11). Furthermore, scholarly work has insufficiently addressed the empirical validation of such index systems through practical application and robust quantitative analysis (12, 13). These limitations not only constrain the practical utility of existing frameworks but also impede evidence-based policymaking and long-term strategic planning for the sector's development (14, 15).

To address these research deficiencies, this study employs a mixed-methods approach combining qualitative and quantitative analysis. The research is structured around several key objectives: (1) to develop a scientifically grounded evaluation index system for China's snow sports development through rigorous methodological procedures; (2) to establish appropriate weighting mechanisms that balance objective data characteristics and normative design principles; (3) to empirically validate the proposed framework through longitudinal analysis of development trends between 2015 and 2019; and (4) to quantify the impact of Winter Olympics investment on different dimensions of snow sports development. By addressing these objectives, this research aims to provide both theoretical contributions to sports development evaluation and practical insights for policymakers and industry stakeholders navigating the post-Olympic development landscape.

## 2 Methods

### 2.1 Expert interview

The interview method can be categorized into structured, unstructured, and semi-structured types based on the degree of control over the interview process (16). This study opted for a combination of unstructured and semi-structured interviews to collect data. The primary reason for this choice was the researchers’ initial uncertainty about which factors might reflect the impact of the 2022 Beijing Winter Olympics on the development of snow sports (17). Consequently, an entirely open attitude was adopted during the initial interviews. As the research progressed, the approach gradually transitioned from unstructured to semi-structured, leading to the development of a semi-structured interview topic outline (Table 1). All interviews were conducted in strict adherence to standard ethical research principles. Prior to participation, each expert was fully informed about the study's purpose and provided verbal consent. To ensure confidentiality and promote candid responses, complete anonymity was guaranteed to all participants; therefore, all data were collected and analyzed without recording any personally identifiable information.

**Table 1:** Semi-structured interview outline.

Serial number	Interview topic
1	Using July 31, 2015, when Beijing successfully bid for the 2022 Winter Olympics as a milestone, please discuss the development overview of snow sports in China before 2015. You may focus on your personal experiences and insights to elaborate on the state of snow sports, especially in the area of recreational skiing, prior to the Olympic bid.
2	Based on your personal experiences and perceptions, discuss the challenges faced by the development of snow sports in China, particularly in the field of recreational skiing, before 2015. What do you believe were the causes of these issues? Follow-up: Which of these development issues were “systemic"? Which were “localized"? Which issues had solutions at that time, and which did not?
3	Based on your personal experiences and insights, please discuss the changes in the development of snow sports in China, particularly recreational skiing, after 2015 compared to before. In what specific ways are these changes manifested? Do you believe these changes are related to the preparations for the 2022 Beijing Winter Olympics? If so, in what ways are they connected? How do you think the Winter Olympics have influenced these “changes"?
4	Based on your personal experiences and perceptions, discuss the challenges faced by the development of snow sports in China, particularly in recreational skiing, after 2015. What do you believe are the causes of these issues? Follow-up: Which issues are longstanding? Which are new challenges arising from changing circumstances? Which issues emerged with the Olympic bid?
5	How do you think the 2022 Beijing Winter Olympics have influenced the development of snow sports in China, especially recreational skiing? In what ways has it impacted snow sports in China (in which aspects)?
6	Based on your previous discussions, from which aspects do you think we should evaluate the development of snow sports in China? In what areas can the development of snow sports in China be reflected? (Or, how can we define the development of snow sports in China?) What is the relationship between these aspects? Have all these aspects been influenced by the successful bid and hosting of the Beijing Winter Olympics? For those affected areas, what are the mechanisms of influence from the Olympics?
7	Do you think the aforementioned aspects can serve as evaluation indicators for the development of snow sports in China? Based on your experience, what indicators are necessary to evaluate the development of snow sports in China, especially recreational skiing?
8	Based on your experience, what challenges might the development of snow sports and recreational skiing in China face in the post-Olympic era? Follow-up: How are these potential issues related to the conclusion of the Winter Olympics?

Currently, qualitative research often employs purposive sampling in interviews, selecting research subjects that can provide rich information based on the research objectives. This method, also known as “theoretical sampling,” aims to capture the internal experiences of the subjects until data saturation is achieved.Unlike probabilistic sampling in quantitative research, it places less emphasis on the number of respondents. In this study, the selection of interview subjects needed to consider the breadth and depth of their understanding of the development of snow sports in China, observing from different perspectives and possessing deep insight. Therefore, based on the “information overload” theory (18), this study selected top experts in the domestic skiing field, using snowball sampling. From March 27 to November 3, 2018, a total of 43 people were interviewed through multiple real-time communication channels such as phone calls, WeChat video, and QQ voice (Table 2).

**Table 2:** Overview of interviewee profiles for snow sports study.

Name	Identity	Data code
Wang * Qiu	Ski Club Manager	01M0327
Wen*Xin	Head of Ski School; Lecturer at China Ski Association	02N0402
Tang*Juan	Influential Ski Broadcaster	03M0407
Meng*Jun	National Snow Sports Team Coach; Beijing Sport University Instructor	04M0409
Liu*Yan	Assistant to Chairman in Ski Resort; Scholar	05M0415
Fa*Ao	Ski Resort General Manager	06N0415
Fu*Yue	Cross-Country Skiing Judge; Scholar	07N0429
Guan*Ming	National Cross-Country Skiing Team Coach	08M0508
Huang*Jing	Ski Resort Executive	09M0516
Gu*Yun	Head of Ice and Snow Campus Activities Enterprise	10M0525
Wang*Dong	National Skiing Judge; Scholar	11M0530
Li*Hui	Ice and Snow Sports Campus Coach; Physical Education Teacher	12M0606
Li*Jun	Winter Sports Center Manager	13N0613
Li*Qian	Winter Olympics Organizing Committee Staff	14M0628
Liu*Hui	Ski Jumping Technical Official; Scholar	15M0705
Li*Ye	Popular Ice and Snow Culture Researcher; Entrepreneur	16M0717
Peng*Yun	Cross-Country Skiing Judge	17N0724
Tian*Nian	Former Secretary-General of China Ski Association	18N0730
Wang*Jiao	Freestyle Skiing World Champion; Scholar	19M0804
Qu*Zhong	Principal of Nanshan Ski School	20M0807
Zhang*Wei	Secretary-General of Beijing Collegiate Sports Association	21N0818
Wang*Chen	Principal of Sunac Ski School	22N0825
Luo*Li	Chairman of Wanlong Paradise Resort	23M0902
Chen*Hong	Chairman of the Board, China Dongxiang (Group) Co., Ltd.	24N0906
Wei*Hua	Chairman of Beijing Antai Snow Industry Investment Management Co., Ltd.	25M0909
Shi*Qiang	Deputy General Manager of Xinjiang Altay Tourism Development Group Co., Ltd.	26M0914
Ma*Ye	General Manager of Changbai Mountain International Ski Resort	27M0919
Chen*Jie	Chairman of Shenyang Strange Slope	28N0925
Wu*Bin	Founder and CEO of Snow Gang Snow Industry	29N0930
Liu*Li	Chairman of Shandong Forest Ski Equipment Co., Ltd.	30M1005
Wang*Song	Chairman of Chongqing Nantian Lake Ski Resort	31M1010
Cheng*Ming	Director of Strategic Development at Cabin Ski Group	32M1018
Wang*Gang	Vice President of Thaiwoo Ski Resort Town	33N1019
Han*Gang	General Manager of Yabuli Sunshine Resort	34M1023
Sun*Qing	Principal of Zhangjiakou Xuanhua No. 2 Middle School	35N1028
Zhang*Tao	CEO of Wanguo Sports	36M1103
Xu*Long	Chairman of EMBA International Alliance	37N1105
Xu*Wen		38M1112
Hao*Hua	Founder of Hao Shihua Ski Training Center	39M1115
Cai*Qiang	Brand Marketing Director of Biancheng Sports Ski Equipment	40N1119
Guo*Dan	Founder of Jupower Sports	41N1124
Zhou*Qian	President of Thaiwoo Ski Resort Town	42M1127
Zhao*Ju	General Manager of Vanke Songhua Lake Resort	43N1130

To efficiently retrieve and analyze interview data, it is stored in coded form (19). The code structure is as follows: the first two digits represent a sequential code, e.g., 17 denotes the 17th interviewee; the third digit represents the interview type, with M indicating an in-person interview and N indicating a non-in-person interview; the last four digits represent the interview date, e.g., 1119 represents November 19, 2018.

To ensure a comprehensive and multi-perspective understanding of China's snow sports development since the successful Olympic bid, a combination of purposive sampling and snowball sampling was employed. Inclusion criteria were: (1) direct involvement in or close observation of key sectors of snow sports (e.g., elite training and administration, recreational ski operations, resort facilities and equipment firms, events and education/training, industry associations and relevant government units); (2) demonstrated breadth and depth of insight through substantial professional experience and/or research; and (3) coverage across major snow sports regions and business types in China. Individuals unable to provide verifiable first-hand practice information or research experience were excluded from the study. To mitigate homogeneity risks inherent in snowballing, referrals were proactively supplemented with experts from different regions and roles (e.g., Northeast–North–Northwest–East–Southwest; state-owned vs. private; venues vs. equipment; education vs. events), while negative case analysis and heterogeneity comparison were employed during coding. Data saturation followed an operational rule of “no new first-level concepts/codes emerging across two consecutive interviews,” documented via MAXQDA2018 memos; after reaching saturation, additional interviews were conducted to confirm saturation stability.

### 2.2 Grounded theory

Scholars such as Hu Jiao (20), Guan Jiang (21), and Zhao De (22), through empirical analysis, have concluded that using the qualitative research method of grounded theory to attribute research problems is a feasible research approach. Grounding is the process of attribution, an effective qualitative method for identifying influencing factors for research problems. The combination of grounding and attribution is significant for enhancing the accuracy and scientific nature of social science research (23). This study combines grounded theory with attribution theory, starting from the collection of individual experience fragments, gradually summarizing the various indicators of the development of snow sports in China, and strives to provide new insights into the study of China's snow sports development through more scientific and rigorous means and methods.

The grounded theory research method, proposed by sociologist B.G. Glaser and others, is a qualitative research method that provides a systematic procedure for analyzing large amounts of qualitative data and forming the theoretical basis of research (24). In qualitative research, scholars adopt different data organization and analysis strategies based on research habits, as introduced in various qualitative research methods in “The SAGE Handbook of Qualitative Research (25). "Professor Chen Xiangming proposed the linear and interactive modes of data analysis, believing that the two can be organically unified (26). Therefore, this study adopts Professor Chen Xiangming's viewpoint, using the grounded theory research method to construct a theoretical framework through bottom-up concept extraction and comparison, and conducting clustering and modification until theoretical saturation is achieved.The specific operations include open coding, axial coding, selective coding, and theory formation.

In this study, MAXQDA 2018 was used for data management and coding, and the implementation was carried out in three progressive stages: “open—main axis—selective”. An initial codebook was established based on several interview texts. The operational definitions were refined through multiple rounds of iterations, and the code evolution and decision-making logic were recorded in memos. To enhance credibility, some of the interview texts were independently reviewed and compared, differences were discussed and resolved, and the codebook and typical anchor attributive paragraphs were subsequently updated. Subsequently, consistent encoding is performed on the remaining texts, and regular comparisons and negative case searches are continuously conducted to ensure logical consistency and conceptual saturation from the data to the categories and main categories.

Coding illustration (excerpt → concept → category → tertiary indicator): Example A (facility expansion): Excerpt—“..upgraded cable cars and chairlifts; added snowmaking machines and snow groomers; invested nearly 300 million RMB..” (05M0415). Open codes: lift upgrades; new snowmaking; new grooming; capital input. Axial category: facility capacity expansion (lifts/magic carpets/snowmaking/grooming). Selective mapping to tertiary indicators (Table 3): “number of aerial lifts/detachable lifts,” “number/length of new and operating magic carpets,” “number of new imported/domestic snowmaking machines,” “number of new imported/domestic (second-hand) snow groomers.”Example B (competitive services and talent): Excerpt—“..assembled outstanding coaches and technical staff; adopted a four-year team leader responsibility system..” (11M0530). Open codes: coaches/technical staff; cross-disciplinary training team; leader responsibility. Axial category: competitive service system and talent development. Tertiary indicators: “number of competitive service personnel,” “number of national team athletes,” and “number of research projects in competitive snow sports.”

**Table 3:** Examples of open coding from interview transcripts.

Concept	Data excerpt	Data source
National Winter Games Training Base/Cable Cars/Chairlifts/Snow making Machines/Snow Groomers/Ski Enterprises/Capital Investment	..Since 2015, the Xinjiang Silk Road Ski Resort has been preparing for the National Winter Games and has become the venue for alpine skiing and the closing ceremony. In 2017, it was designated as a National Training Base and planned to establish a Snow Sports Branch of Beijing Sport University (not yet implemented). Subsequently, the ski resort underwent extensive infrastructure development, investing nearly 300 million to renovate the ski equipment hall and upgrade cable car and chairlift facilities. Before 2018, the Silk Road Ski Resort dominated Xinjiang, but then the Jiangjun Mountain Ski Resort and Koktokay Ski Resort in Altay quickly emerged as strong competitors, both making significant investments and achieving good results..	05M0415
Coaches/Technical Staff/Team Leaders	..Following the successful Olympic bid, China assembled outstanding coaches and technical staff to jointly select and test athletes. The selected elite athletes underwent intensive training, forming a cross-disciplinary training team. In preparation for the Beijing Olympics, a four-year cycle team leader responsibility system was adopted, with each leader guiding their team according to project needs to ensure team stability and sustainable development..	11M0530

#### 2.2.1 Open coding (level one)

Open coding is the initial stage of the grounded theory analysis method, aiming to find localized concepts and understand the way researchers view the world through data fragmentation, concept assignment, and recombination (27). This process usually occurs simultaneously with data entry, extracting concepts and categories from textual data.This study uses MAXQDA2018 qualitative research software to analyze 43 interview materials, with Table 3 showing some of the text coding.

Through the analysis of 43 interview materials, using the Beijing Winter Olympics’ impact on the development of snow sports as a clue and following the principles of open coding, a total of 184 concepts were extracted from the raw data (Table 4).

**Table 4:** Key concepts extracted from winter olympics impact on snow sports.

Concept 1	Concept 2	Concept 3
Winter Olympics Champion	Financing of Listed Snow Sports Companies	Club Founding Time
Ski Enterprise Marketing Model	Advertising Sponsorship	Composition of Simulator Skiing Participants
Equity Distribution of Listed Snow Sports Companies	Training Market Policies	Distribution of Dry Skiing Locations
Ski WeChat Public Account Views	Competition Frequency	Indoor Ski Resort Opening Hours
Event Hosting Costs	Types of Ski Resorts with Aerial Cableways	World Championship Events
Daily Dry Skiing Participants	World Cup Ranking	Ski Resort Magic Carpet Length
National Social Science Fund	Number of Ski Enterprises	Market Value of Listed Snow Sports Companies
Distribution of Skiers	Once a Year	Simulator Skiing Duration
Dry Skiing Time Distribution	Points	FIS-Certified Slope Locations
World Cup Events	Indoor Ski Resort Scale	New Media Communication Channels
New Imported Snowmaking Machines	Event Hosting Costs	Number of Snowmaking Machines in Ski Resorts
Search Frequency	Equipment Market Brands	Event Scale
Outdoor Ski Resorts	Winter Olympic Athlete Performance	Number of Domestic Detachable Cableways
Points	Age Distribution of Domestic Ski Tourists	World Cup Athlete Performance
Ski WeChat Public Account Shares	Twin Tip Binding Brands	Ski Resort Opening Hours for Terrain Parks
Assessment Methods for Ski Social Sports Instructors	Types of Snow Sports Clubs	Nature of Participation
Nature of Work for Competition Service Staff	Number of Domestic Second-Hand Snow Groomers	Ski Simulator Prices
Technological Content of Equipment Market	Number of Imported Second-Hand Snow Groomers	Number of New Ski Resorts
Scale of National Snow Sports Training Bases	Distribution of Ski Simulators	New Magic Carpet Installation Time
Categories of National Team Athletes	Work Attributes of Ski Enterprise Employees	Names of FIS-Certified Slopes
Club Personnel Structure	Legal Capital	Distribution of Dry Ski Resorts
Performance Levels of Winter Olympic Athletes	Scale of World Championship Teams	Assessment Standards for Ski Social Sports Instructors
Holiday Skiing Participants	Length of FIS-Certified Slopes	Ski Equipment Rental Situation
Faculty Strength of Ice and Snow Sports Specialty Schools	Competition Events	Scale of Winter Olympic Teams
Club Scale	Holiday Skiing Participants	Age Distribution of Skiers
Event Industry Chain	Levels of Competitive Snow Sports Topics	Scale of Ski Enterprises
Snow Groomer Prices	Number of Companies Investing in Ski Enterprises	Distribution of Simulator Skiing Times
Major Season	Total Social Security Contributions by Ski Enterprises	Types of Competitive Snow Sports Topics
Length of New Magic Carpets	Countries Importing Twin Tip Bindings	Investment in Magic Carpets
Training Prices	Names of Domestic Ski Enterprises	Job Content of Competition Service Staff
Categories of Domestic Ski Tourism Consumption	Number of Event Spectators	Research Directions of Competitive Snow Sports Topics
Nature of the Competition	Scale of Dry Skiing Venues	Revenue Situation
Total Capital Stock of Listed Snow Sports Companies	Level Distribution of Ski Social Sports Instructors	Equity Distribution of Listed Snow Sports Companies
New Domestic Snowmaking Machines	Search Time	Scale of Ski Resorts with Detachable Cableways
Scale of Ski Resorts with Aerial Cableways	Number of Event Spectators	Athletic Performance of Universities Offering Ice and Snow Sports Majors
Level of Financial and Policy Support for Schools	Distribution of Snow Sports Training Base Locations	Income Situation of Skiers
Ski Equipment Rental Situation	Distribution of Universities Offering Ice and Snow Sports Majors	Search Content
Distribution of Ski Resorts with Detachable Cableways	Daily Skiing Participants	Skiing Frequency
Professional Competence of Training Personnel	Scale of Dry Ski Resorts	Snowmaking Machine Prices
Per Capita Income Level	World Championship Ranking	Event IP
Founding Time of Ski Enterprises	Number of Followers of Ski WeChat Public Accounts	Distribution of Participants
Skiing Duration	Professional Competence of Ski Social Sports Instructors	Number of New Magic Carpets
Number of Major Events Hosted	Elevation Difference of FIS-Certified Slopes	Competition Events
Company Restructuring	Hosting Organization	Competition Results of Ice and Snow Sports Specialty Schools
Browsing Categories	Daily Skiing Participants	Number of Imported Detachable Cableways
Number of Employees in Ski Enterprises	Company Acquisition	Event Categories
Distribution of Ice and Snow Sports Specialty Schools	Passenger Traffic of Ski Trains	Financing Scale of Ski Enterprises
Performance of National Team Athletes	Names of Foreign Ski Enterprises	Ski Simulator Brands
Holiday Dry Skiing Participants	Founding Time of Ice and Snow Sports Specialty Schools	Training Methods
Number of Posts on Ski WeChat Public Accounts	Establishment of Training Bases	Performance of World Championship Athletes
Browsing Time	Distribution of Simulator Skiing Locations	Types of Training Markets
Types of Equipment Markets	Types of Ski WeChat Public Accounts	Scale of World Cup Teams
Age Classification of National Team Athletes	Distribution of Ski Resort Magic Carpets	Level of World Championship Athletes
Distribution of National Team Athletes	Level of World Cup Athletes	Categories of Universities Offering Ice and Snow-Related Majors
Length of Aerial Cableways	Distribution of Domestic Ski Tourism Locations	Skiing Duration
Ice and Snow Majors in Universities	Service Projects of Competition Service Staff	Once Every Two Years
Categories of Ice and Snow Sports Specialty Schools	Distribution of Domestic Ski Resorts	Demand for Twin Tip Bindings
Indoor Ski Resorts	Average Passenger Traffic of Ski Resorts	Distribution of Indoor Ski Resorts
Club Profit Models	Winter Olympics Ranking	Event Influence
Number of Snow Groomers in Ski Resorts	Dry Ski Resorts	Consumption Concepts
Hosting Location	Countries Importing Ski Simulators	Profit of Listed Snow Sports Companies

#### 2.2.2 Axial coding (secondary level)

**Figure 1:**
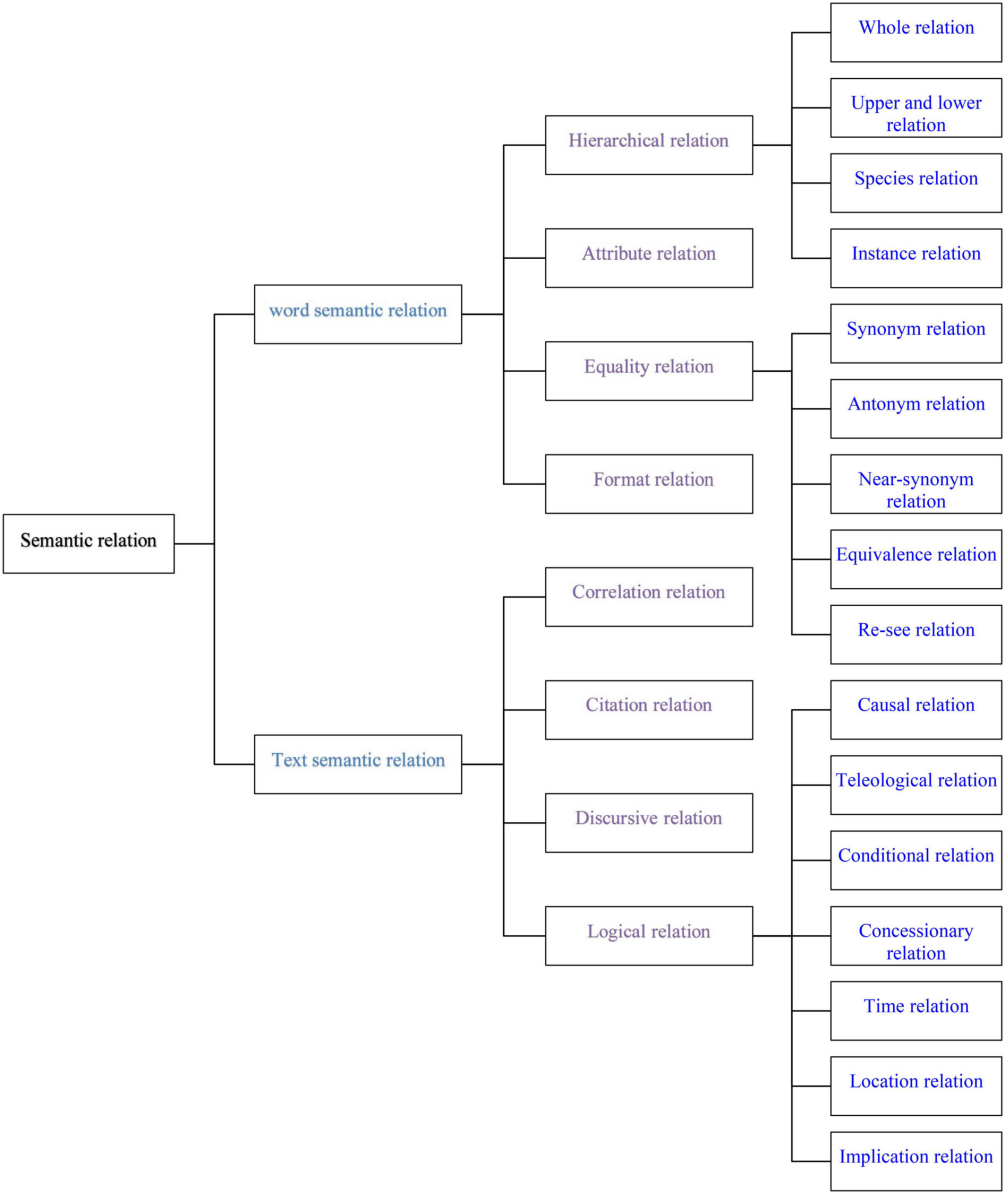
Framework of semantic relationships in grounded theory analysis.

Axial coding primarily involves further clustering the concepts obtained from the initial coding, establishing connections between categories based on relationships such as similarity, process, and subject.In this study, the concepts and their inherent and logical relationships were continuously compared and classified by referencing the model established by semantic segmentation scholar Wang Zhijin (28) (Figure 1).

By carefully analyzing and categorizing the 184 concepts obtained through open coding, 38 categories were ultimately formed, which serve as the primary indicators of the development of snow sports (Table 5).

**Table 5:** Categorized impacts of Beijing winter olympics on snow sports development.

Serial number	Category
1	Winter Olympics Snow Sports Performance
2	Snow Sports World Championship Performance
3	Snow Sports World Cup Performance
4	Number of Ski Events (Professional/Public Competitions)
5	Number of Event Parameter Units
6	Number of National Team Athletes
7	Number of Competitive Service Personnel
8	Number of National Snow Sports Training Bases Accredited by the Winter Sports Center
9	Number of Research Projects in Competitive Snow Sports
10	Number of FIS-Certified Slopes in Chinese Ski Resorts
11	Number of Skiing Sessions on Simulators
12	Number of Dry Skiing Sessions
13	Number of Skiers (Ski Resort Visits, Annual Average Skiing Frequency)
14	Number of Ski Social Sports Instructors
15	Number of Ski Enterprise Employees Paying Social Security
16	Number of Domestic Ski Resorts
17	Total Number of Aerial Cableways in Ski Resorts (Number of Ski Resorts with Aerial Cableways)
18	Number of Ski Resorts with Detachable Cableways (Imported/Domestic, Total)
19	Total Number of Operating Magic Carpets in Ski Resorts (Number of New Magic Carpets)
20	Total Length of Operating Magic Carpets in Ski Resorts (Length of New Magic Carpets)
21	Number of New Second-Hand Snow Groomers in Ski Resorts (Imported/Domestic)
22	Number of New Snowmaking Machines in Ski Resorts (Imported/Domestic)
23	Number of Indoor Ski Resorts
24	Dry Ski Resorts (Number, Area)
25	Number of Ski Simulators
26	Import Volume of Twin Tip Bindings (Detachers)
27	Number of Ski Enterprises
28	Snow Sports Clubs
29	Number of Nationally Accredited Ice and Snow Sports Specialty Schools
30	Number of Universities Offering Ice and Snow-Related Majors
31	Baidu Search Index
32	Comprehensive Communication Index (WCI) of Leading Ski WeChat Public Accounts
33	Registered Capital of Ski Enterprises
34	Per Capita Consumption in Domestic Ski Tourism
35	Scale of the Equipment Market
36	Scale of the Training Market
37	Scale of the Event Market
38	Listed Snow Sports Companies (P/E Ratio, Market Value, Stock Price)

#### 2.2.3 Selective coding (tertiary level)

**Figure 2:**
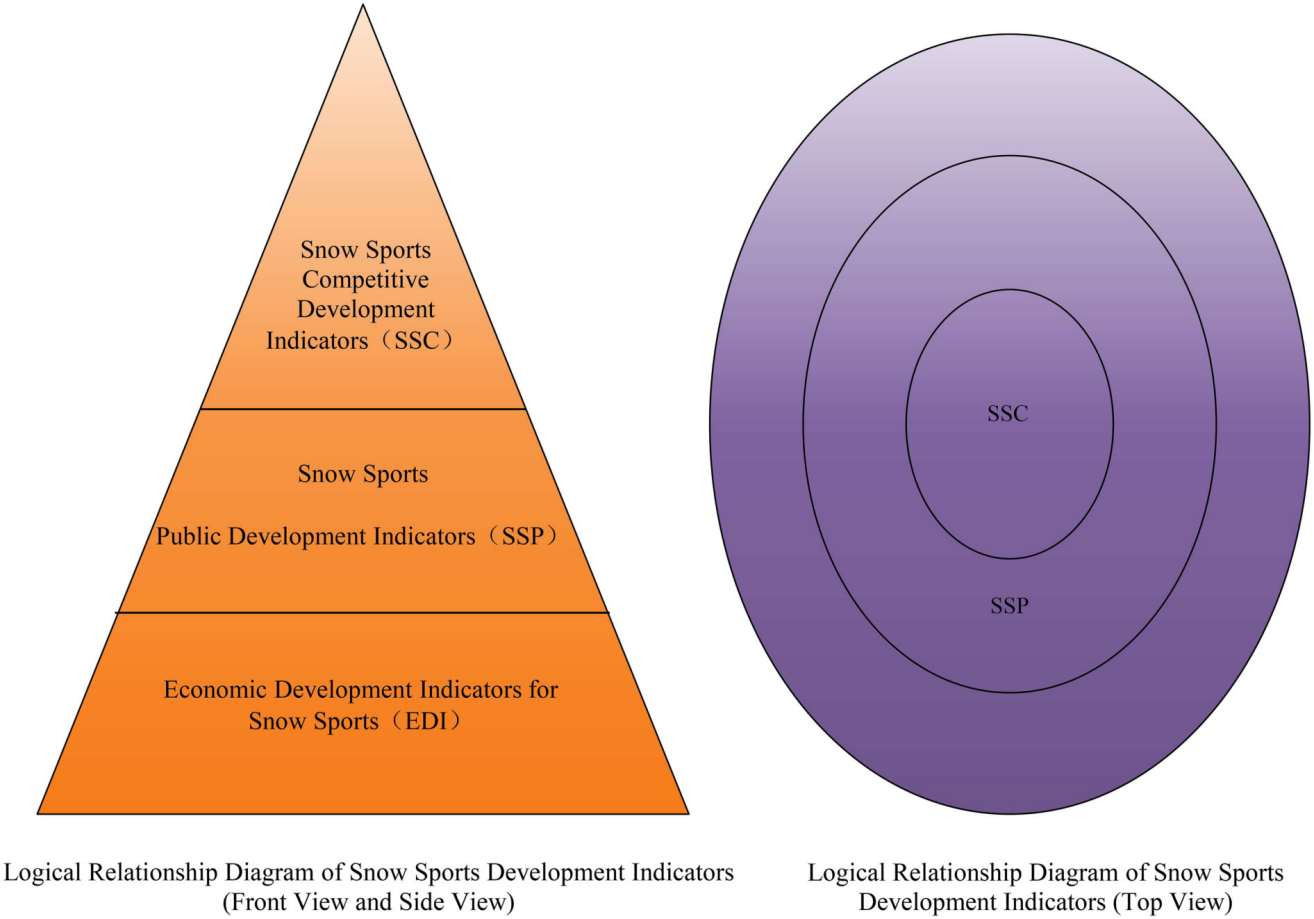
Concentric circle model of snow sports development indicators.

Selective coding refers to the process of choosing a core category and systematically linking all other categories to this core category (Strauss & Corbin) (29). This stage of the coding process integrates all previous analytical work, analyzing the associations between the core or main category and other categories.Data is grouped to facilitate the identification of patterns and trends when observing specific dimensions within the categories.In this study, continuous comparison was conducted using memos and original data, with “development of snow sports” as the core category. All main categories and categories were integrated to develop the theoretical model as shown in Figure 2.

In constructing the development indicator system for snow sports, this study reclassified the indicators, considering the potential overlaps and intersections in the traditional fourfold classification of sports in China (competitive sports, mass sports, school sports, and sports industry) in practical application. Given the unique nature of snow sports, school sports are regarded as part of mass sports, while the sports industry and mass sports overlap in practical work. Therefore, this study divides the indicators into three aspects: “competitive development indicators for snow sports,” “mass development indicators for snow sports,” and “economic development indicators for snow sports.”

The logical relationship of the snow sports development indicators forms a “conical” structure in three-dimensional space, appearing as a “pyramid” shape from the front and side views. Competitive sports are at the “pinnacle,” representing the highest level of snow sports in China; mass sports form the “body” of the pyramid, supporting the height of competitive sports through widespread participation; the development of the sports industry is at the “base,” providing economic support. From a top view, the indicator system presents a “concentric circle” layered pattern, with competitive sports development indicators at the core, mass sports development indicators in the middle layer, and economic development indicators on the periphery.

This logical relationship indicates that the development of snow sports requires the leadership of competitive sports, the popularization of mass sports, and the economic support of the sports industry. The level of development in competitive sports directly reflects the competitive level of a project, the degree of popularization in mass sports indicates the sustainability and social impact of a project, and the development of the sports industry provides the necessary economic foundation and resource assurance for snow sports.

The integration pathway is structured as follows: semi-structured interviews are first conducted to elicit first-hand experiential factors; open, axial, and selective coding based on grounded theory is then applied to derive a three-level indicator system; combined weighting methods are used to obtain quantifiable weights; multi-source data from 2015 to 2019 are employed to compute indicators and synthesize development indices; panel fixed-effects and random-effects regressions are performed to test external validity and impact mechanisms; finally, empirical results provide feedback to refine indicator interpretations and weight explanations, forming a closed methodological loop.

### 2.3 Index system construction

Based on attribution theory and employing a research paradigm that combines interviews and grounded theory, this study interviewed leading experts and scholars in the field of snow sports in China to obtain firsthand information on the development of snow sports since the bid for the 2022 Beijing Winter Olympics. Through coding analysis of this data, an evaluation indicator system for the development of snow sports in China was established, consisting of 54 tertiary indicators, 13 secondary indicators, and 3 primary indicators (Table 6).

**Table 6:** Structure of snow sports evaluation indicator system.

Primary Indicator	Secondary Indicator	Tertiary Indicator
Competitive Development Indicators for Snow Sports	Development Indicators for Competitive Achievements in Snow Sports	Number of Medals in Snow Sports at the Winter Olympics
		Number of Medals in Snow Sports at the World Championships
		Number of Medals in Snow Sports at the World Cup
	Development Indicators for Snow Sports Events	Number of Ski Events
		Professional Competitions
		Mass Competitions
		Number of Event Parameter Units
	Development Indicators for Competitive Talent in Snow Sports	Number of National Team Athletes
		Number of Competitive Service Personnel
	Development Indicators for Competitive Services in Snow Sports	Number of National Snow Sports Training Bases Recognized by the Winter Sports Center
		Number of Research Projects Related to Competitive Snow Sports
		Number of FIS-Certified Ski Slopes in China
Mass Development Indicators for Snow Sports	Development Indicators for Snow Sports Participation (Demand)	Number of Ski Visits to Ski Resorts (Unit: 10,000 Visits)
		Number of Ski Visits on Ski Simulators (Unit: 10,000 Visits)
		Number of Ski Visits on Dry Slopes (Unit: 10,000 Visits)
		Number of Skiers
		Annual Average Skiing Frequency per Person
	Mass Service Personnel in Snow Sports (Supply)	Number of Ski Social Sports Instructors
		Number of Employees in Ski Enterprises Paying Social Insurance
	Development Indicators for Snow Sports Facilities (Concrete Platform)	Number of Ski Resorts in China
		Number of Ski Resorts with Aerial Lifts
		Total Number of Aerial Lifts in Ski Resorts
		Number of Ski Resorts with Detachable Lifts (Tows)
		Total Number of Detachable Lifts (Tows)
		Number of Imported Detachable Aerial Lifts (Tows)
		Number of Domestic Detachable Aerial Lifts (Tows)
		Number of New Magic Carpets in Ski Resorts
		Length of New Magic Carpets in Ski Resorts (Unit: Kilometers)
		Total Number of Operating Magic Carpets in Ski Resorts
		Total Length of Operating Magic Carpets in Ski Resorts (Unit: Kilometers)
		Number of New Imported Snow Groomers in Ski Resorts
		Number of New Domestic Snow Groomers in Ski Resorts
		Number of New Imported Used Snow Groomers in Ski Resorts
		Number of New Imported Snowmaking Machines in Ski Resorts
		Number of New Domestic Snowmaking Machines in Ski Resorts
		Number of Indoor Ski Resorts
		Number of Dry Ski Resorts
		Area of Dry Ski Slopes (Unit: 10,000 Square Meters)
		Number of Ski Simulators
		Import Volume of Alpine Bindings (Releasers) (Customs Data, Unit: Tons)
	Development Indicators for Snow Sports Organizations	Import Volume of Alpine Bindings (Releasers) (Customs Data, Unit: Tons)
		Snow Sports Clubs
		Number of Nationally Recognized Ice and Snow Sports Specialty Schools
		Number of Universities Offering Ice and Snow Sports-Related Majors in China
	Development Indicators for Snow Sports Communication	Baidu Search Index
		Comprehensive Communication Index of Leading Ski WeChat Public Accounts (WCI)
Economic Development Indicators for Snow Sports	Development Indicators for Investment in Snow Sports Projects	Registered Capital of Ski Enterprises
	Development Indicators for Snow Sports Consumption	Per Capita Consumption in Domestic Ski Tourism
	Development Indicators for Snow Sports Market Size	Equipment Market Size (Unit: 100 Million RMB)
		Training Market Size (Unit: 100 Million RMB)
		Event Market Size (Unit: 100 Million RMB)
	Development Indicators for Listed Snow Sports Companies	Price-to-Earnings Ratio of Listed Snow Sports Companies
		Market Value of Listed Snow Sports Companies
		Stock Price of Listed Snow Sports Companies

### 2.4 Data sources

Currently, the statistical data on snow sports in China is still insufficient. Therefore, to enhance related research, this study collects snow sports data from multiple sources. Since the data released at the end of 2021 pertains to 2020, and some data collected at the end of the year has not yet been published, this study selects the time span of 2015–2019 for relevant data. Data sources include the “China Ski Industry Core Data Report,” the “Beijing 2022 Winter Olympics Bid Report,” Qichacha, and some data obtained through surveys and interviews.

The data from 2015 to 2019 primarily derive from the following sources: (1) The “Core Data Report on China's Ski Industry” and related annual industry reports, which provide information on facility-related indicators such as the number of ski resorts, cable cars, magic carpets, snowmaking and grooming equipment, as well as indoor and dry ski facilities; (2) The “Beijing 2022 Winter Olympics Bid Report” and publicly available preparatory documents, which serve as references for investment standards associated with the event and its infrastructure development; (3) Enterprise registration and business credit databases, including Qichacha, which are used to obtain data on registered capital and employee numbers of skiing-related enterprises; (4) Estimates derived from industry research and survey questionnaires, with consistent methodological calibration applied across time periods. Potential limitations include discrepancies in timing or definitions across sources, underreporting by small and medium-sized enterprises, and regional variations in statistical practices. These issues have been mitigated through cross-verification using multiple data sources, year-on-year trend analysis, and outlier identification via longitudinal tracking.

### 2.5 Combined weighting method determination

The combined weighting approach integrated both objective data characteristics and subjective design considerations (30). The coefficient of variation method was first employed as an objective weighting method, calculated as follows:

Vi=σi/μ (1)

Wi=Vi/ΣVi (2)

where *μ* represents the mean of the indicator data, *σ* denotes the standard deviation, Vi denotes the coefficient of variation, and Wi represents the normalized weight. Subsequently, equal weighting was applied to reflect the normative design principle of equal dimensional importance (Table 7). The final combined weight was determined as the arithmetic mean of the weights derived from both the coefficient of variation method and the equal weighting method, as detailed in Table 8.

**Table 7:** Combined weights for tertiary indicators in snow sports evaluation.

Tertiary indicator	Mean	Standard deviation	Coefficient of variation	Combined weight under coefficient of variation and equal weight
Number of Snow Sports Medals at the Winter Olympics	3.8333	1.9408	0.5063	0.0250
Number of Snow Sports Medals at the World Championships	5.1667	1.7224	0.3334	0.0165
Number of Snow Sports Medals at the World Cup	12.3333	7.2019	0.5839	0.0288
Number of Ski Events	202.0000	86.6695	0.4291	0.0212
Professional Competitions	56.0000	15.9750	0.2853	0.0141
Public Competitions	110.1667	41.7201	0.3787	0.0187
Number of Event Parameter Units	117.0000	43.1555	0.3689	0.0182
Number of National Team Athletes	793.0000	199.4813	0.2516	0.0124
Number of Competitive Service Personnel	5,403.3333	1,523.2608	0.2819	0.0139
Number of National Snow Sports Training Bases Accredited by the Winter Sports Center	4.5000	3.0822	0.6849	0.0338
Number of Research Projects Related to Competitive Snow Sports	20.1667	8.4479	0.4189	0.0207
Number of FIS-Certified Slopes in Chinese Ski Resorts	19.3333	2.2509	0.1164	0.0057
Number of Ski Resort Visits	1,643.0000	351.0983	0.2137	0.0106
Number of Ski Simulator Visits	42.4950	36.4468	0.8577	0.0424
Number of Dry Ski Visits	19.1333	15.0834	0.7883	0.0389
Number of Skiers	1,163.1667	142.6007	0.1226	0.0061
Annual Average Skiing Frequency	1.4500	0.1378	0.0951	0.0047
Number of Ski Social Sports Instructors	654.6667	253.1242	0.3866	0.0191
Number of Ski Enterprise Employees Paying Social Security	69,846.3333	39,878.8807	0.5710	0.0282
Number of Domestic Ski Resorts	690.6667	73.0963	0.1058	0.0052
Number of Ski Resorts with Aerial Cableways	140.3333	19.3770	0.1381	0.0068
Total Number of Aerial Cableways in Ski Resorts	233.6667	37.6493	0.1611	0.0080
Number of Ski Resorts with Detachable Cableways	17.8333	6.0139	0.3372	0.0167
Total Number of Detachable Cableways	49.1667	16.7382	0.3404	0.0168
Number of Imported Detachable Aerial Cableways	34.5000	7.5565	0.2190	0.0108
Number of Domestic Detachable Aerial Cableways	14.0000	8.2704	0.5907	0.0292
Number of New Magic Carpets in Ski Resorts	173.6667	45.6844	0.2631	0.0130
Length of New Magic Carpets in Ski Resorts	25.4667	6.3767	0.2504	0.0124
Total Number of Operating Magic Carpets in Ski Resorts	1089.1667	312.5664	0.2870	0.0142
Total Length of Operating Magic Carpets in Ski Resorts	159.4000	43.2739	0.2715	0.0134
Number of New Imported Snow Groomers in Ski Resorts	56.6667	14.1091	0.2490	0.0123
Number of New Domestic Snow Groomers in Ski Resorts	22.3333	8.3106	0.3721	0.0184
Number of New Imported Second-Hand Snow Groomers in Ski Resorts	6.6667	3.9328	0.5899	0.0291
Number of New Imported Snowmaking Machines in Ski Resorts	693.1667	200.7131	0.2896	0.0143
Number of New Domestic Snowmaking Machines in Ski Resorts	277.1667	198.8853	0.7176	0.0354
Number of Indoor Ski Resorts	24.6667	8.8468	0.3587	0.0177
Number of Dry Ski Resorts	31.5000	17.5357	0.5567	0.0275
Area of Dry Ski Facilities	12.6550	5.3662	0.4240	0.0209
Number of Ski Simulators	182.8333	196.7897	1.0763	0.0532
Twin Tip Binding Imports (Customs Data, Unit: Tons)	119.0033	19.9740	0.1678	0.0083
Number of Ski Enterprises	2,952.5000	86.9615	0.0295	0.0015
Number of Snow Sports Clubs	890.6667	384.9185	0.4322	0.0213
Number of Ice and Snow Sports Specialty Schools	36.3333	20.0765	0.5526	0.0273
Number of Universities Offering Ice and Snow-Related Majors	19.3333	8.6178	0.4457	0.0220
Baidu Search Index	466,754.6667	70,765.2548	0.1516	0.0075
Comprehensive Communication Index (WCI) of Leading Ski WeChat Public Accounts	1,734.1667	345.2080	0.1991	0.0098
Registered Capital of Ski Enterprises	111469762788.7750	28622644604.2888	0.2568	0.0127
Per Capita Consumption in Domestic Ski Tourism	5,551.6667	901.3953	0.1624	0.0080
Scale of the Equipment Market	91.7833	30.3915	0.3311	0.0164
Scale of the Training Market	63.4167	7.5372	0.1189	0.0059
Scale of the Event Market	81.0000	29.3752	0.3627	0.0179
Price-to-Earnings Ratio of Listed Snow Sports Companies	923.4233	98.0648	0.1062	0.0052
Market Value of Listed Snow Sports Companies	5,905.9233	9,059.7068	1.5340	0.0758
Stock Price of Listed Snow Sports Companies	39.2100	4.9882	0.1272	0.0063

**Table 8:** Hierarchical weight system for snow sports development indicators.

Primary indicator	Primary weight	Secondary indicator	Secondary weight	Tertiary indicator	Tertiary weight	Initial weight
Snow Sports Competitive Development Indicators	0.3333	Snow Sports Competitive Performance Development Indicators	0.3069	Number of Snow Sports Medals at the Winter Olympics	0.3556	0.0250
				Number of Snow Sports Medals at the World Championships	0.2342	0.0165
				Number of Snow Sports Medals at the World Cup	0.4102	0.0288
		Snow Sports Event Development Indicators	0.3151	Number of Ski Events	0.2935	0.0212
				Professional Competitions	0.1951	0.0141
				Public Competitions	0.2591	0.0187
				Number of Event Parameter Units	0.2523	0.0182
		Development Indicators for Competitive Talent in Snow Sports	0.1150	Number of National Team Athletes	0.4715	0.0124
				Number of Competitive Service Personnel	0.5285	0.0139
		Development Indicators for Competitive Services in Snow Sports	0.2630	Number of National Snow Sports Training Bases Accredited by the Winter Sports Center	0.5613	0.0338
				Number of Research Projects Related to Competitive Snow Sports	0.3433	0.0207
				Number of FIS-Certified Slopes in Chinese Ski Resorts	0.0954	0.0057
Snow Sports Public Development Indicators	0.3333	Population Participation Development Indicators for Snow Sports (Demand)	0.1647	Number of Ski Resort Visits	0.1029	0.0106
				Number of Ski Simulator Visits	0.4129	0.0424
				Number of Dry Ski Visits	0.3795	0.0389
				Number of Skiers	0.0590	0.0061
				Annual Average Skiing Frequency	0.0458	0.0047
		Public Service Personnel in Snow Sports (Supply)	0.0759	Number of Ski Social Sports Instructors	0.4038	0.0191
				Number of Ski Enterprise Employees Paying Social Security	0.5962	0.0282
		Development Indicators for Snow Sports Facilities (Concrete Platforms)	0.6158	Number of Domestic Ski Resorts	0.0136	0.0052
				Number of Ski Resorts with Aerial Cableways	0.0178	0.0068
				Total Number of Aerial Cableways in Ski Resorts	0.0207	0.0080
				Number of Ski Resorts with Detachable Cableways	0.0434	0.0167
				Total Number of Detachable Cableways	0.0438	0.0168
				Number of Imported Detachable Aerial Cableways	0.0282	0.0108
				Number of Domestic Detachable Aerial Cableways	0.0761	0.0292
				Number of New Magic Carpets in Ski Resorts	0.0339	0.0130
				Length of New Magic Carpets in Ski Resorts	0.0322	0.0124
				Total Number of Operating Magic Carpets in Ski Resorts	0.0370	0.0142
				Total Length of Operating Magic Carpets in Ski Resorts	0.0350	0.0134
				Number of New Imported Snow Groomers in Ski Resorts	0.0321	0.0123
				Number of New Domestic Snow Groomers in Ski Resorts	0.0479	0.0184
				Number of New Imported Second-Hand Snow Groomers in Ski Resorts	0.0760	0.0291
				Number of New Imported Snowmaking Machines in Ski Resorts	0.0373	0.0143
				Number of New Domestic Snowmaking Machines in Ski Resorts	0.0924	0.0354
				Number of Indoor Ski Resorts	0.0462	0.0177
				Number of Dry Ski Resorts	0.0717	0.0275
				Area of Dry Ski Facilities	0.0546	0.0209
				Number of Ski Simulators	0.1386	0.0532
				Twin Tip Binding Imports (Customs Data, Unit: Tons)	0.0216	0.0083
		Organizational Development Indicators for Snow Sports	0.1158	Number of Ski Enterprises	0.0163	0.0015
				Number of Snow Sports Clubs	0.2387	0.0213
				Number of Ice and Snow Sports Specialty Schools	0.3052	0.0273
				Number of Universities Offering Ice and Snow-Related Majors	0.2462	0.0220
		Communication Development Indicators for Snow Sports	0.0278	Baidu Search Index	0.4323	0.0075
				Comprehensive Communication Index (WCI) of Leading Ski WeChat Public Accounts	0.5677	0.0098
Economic Development Indicators for Snow Sports	0.3333	Investment Development Indicators for Snow Sports Projects	0.0857	Registered Capital of Ski Enterprises	1.0000	0.0127
		Consumption Development Indicators for Snow Sports	0.0540	Per Capita Consumption in Domestic Ski Tourism	1.0000	0.0080
		Market Scale Development Indicators for Snow Sports	0.2710	Scale of the Equipment Market	0.5634	0.0164
				Scale of the Training Market	0.2022	0.0059
				Scale of the Event Market	0.6171	0.0179
		Development Indicators for Listed Snow Sports Companies	0.5893	Price-to-Earnings Ratio of Listed Snow Sports Companies	0.0601	0.0052
				Market Value of Listed Snow Sports Companies	0.8679	0.0758
				Stock Price of Listed Snow Sports Companies	0.0720	0.0063

### 2.6 Calculation and evaluation

**Figure 3:**
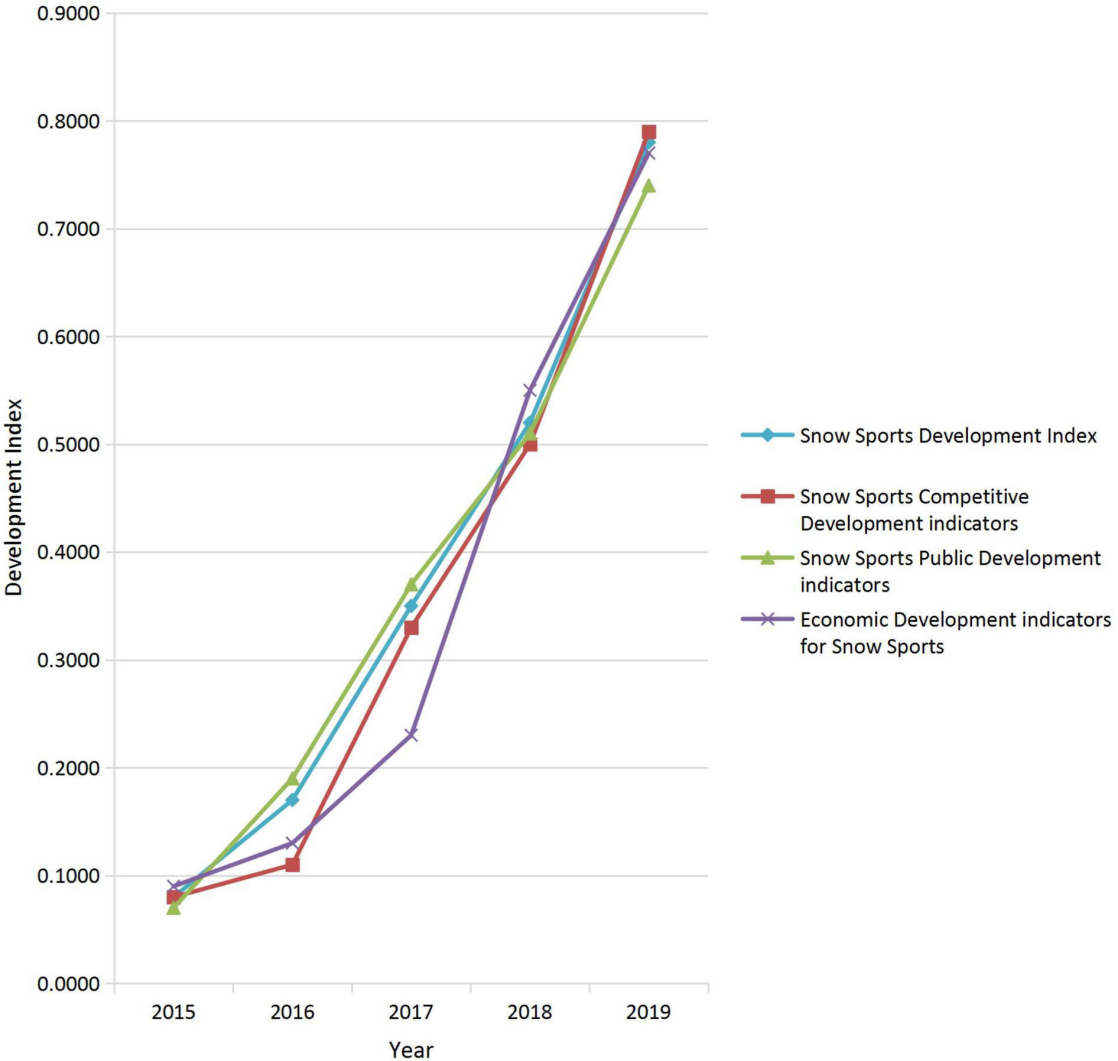
Development of snow sports in China from 2015 to 2019.

The development index of snow sports in China was calculated using the aforementioned methods, and the specific results are shown in Table 9. As shown in Table 9 and Figure 3, the development of snow sports in China has been rapid. From 2015 to 2019, the overall snow sports development index rose from 0.0705 to 0.7704 (10.92 times). The competitive development index increased from 0.0745 to 0.7129 (10.64 times), the mass (public) development index from 0.0576 to 0.7323 (12.71 times), and the economic development index from 0.0869 to 0.7160 (8.82 times). These results suggest substantial progress across all dimensions, with the fastest growth observed in mass participation. However, due to geographical and climatic factors, the economic benefits of snow sports have increased relatively slowly, especially in tropical and subtropical provinces, where high development costs have prevented full realization of economic effects. Nonetheless, the overall development speed of snow sports in China remains highly remarkable.

**Table 9:** Development Index of snow sports and Its dimensions in China, 2015–2019.

Year	Snow sports development index	Competitive snow sports development	Public snow sports development	Economic development of snow sports
2015	0.0705	0.0745	0.0576	0.0869
2016	0.1694	0.1144	0.1952	0.1350
2017	0.3510	0.3236	0.3747	0.2289
2018	0.4990	0.5012	0.5158	0.5409
2019	0.7704	0.7129	0.7323	0.7160
Growth Rate	10.9245	10.6421	12.7116	8.8152

## 3 Results

### 3.1 Variable selection

This study primarily investigates the impact of the Beijing Winter Olympics on the development of snow sports in China. Therefore, the dependent variable is the development level of snow sports in China, represented by a snow sports development index; the key independent variable is the level of investment in the Beijing Winter Olympics (Input). The investment in the Beijing Winter Olympics encompasses various aspects, many of which are intangible assets that cannot be specifically quantified. Therefore, the primary core investment—economic investment—is used as a substitute variable for the level of investment in the Beijing Winter Olympics.

The control variables selected are economic development level (Economy), degree of openness (Open), level of fiscal expenditure (Finance), and cost expenditure (Cost). The reasons for variable selection are as follows: (1) The level of economic development is the foundation for high-quality development of a country and region, and it plays a crucial supporting role in the development of snow sports.This is not only reflected in the construction of snow sports infrastructure but also in the increased consumer willingness to participate in snow sports as their purchasing power grows. Therefore, the level of economic development significantly impacts the growth of snow sports (31). (2) Regions with advanced snow sports development are primarily in Europe and North America. Further expansion of openness will facilitate greater interaction between China's snow sports sector and the international community, potentially leading to a more extensive snow sports industry trade chain and having a positive impact on snow sports development (32). (3) The level of fiscal expenditure determines the development progress of snow sports infrastructure, which is critically linked to the development of snow sports and the broader ice and snow sports sector (33). (4) The long-term and efficient development of snow sports largely depends on cost issues. Currently, high costs are one of the primary barriers to the development of snow sports. For businesses operating in snow sports, achieving sustained profitability is a prerequisite for long-term operations. Excessive operating costs may result in prolonged losses for companies (34), and if government subsidies are withdrawn, the entire industry could face significant operational challenges. Therefore, cost expenditure is a critical factor affecting the development of snow sports (35).

### 3.2 Model specification

Based on the multidimensional panel data of snow sports development, this study employs a fixed effects model for estimation, as specified in Equation 3 (36, 37). In this model, Snowsport i, t represents the level of snow sports development, Input i, t denotes the level of investment in the Winter Olympics, Control i, t indicates the control variables, μ i represents the individual effects, *σ*t denotes the time effects, and *ε* it represents the random disturbance term. Subsequently, to verify the stability of the empirical results, this study will conduct robustness checks to ensure the reasonableness and scientific validity of the results.

Snowsporti,t=α+β1Inputi,t+ΣβiControli,t+μi+σt+εit (3)

### 3.3 Empirical outcomes

The overall empirical test results of the impact of Winter Olympics investment on the development of snow sports are shown in Table 10. The regression coefficient for Input is 0.1661, which is significantly positive at the 10% level; the regression coefficient for Economy is 0.1593, which is not significant; the regression coefficient for Open is 0.0555, which is significant at the 10% level; the regression coefficient for Finance is 0.0197, which is not significant; the regression coefficient for Cost is −0.0190, which is significantly negative at the 5% level; the regression coefficient for the constant term is −1.8988, which is significant at the 1% level, with a goodness-of-fit of 0.7402. From the regression results, it can be concluded that the model fits well, and the investment in the Winter Olympics has a significant positive impact on the development of snow sports, enhancing its development level. Among the control variables, the level of economic development has a limited direct promoting effect on the development of snow sports. In contrast, the level of openness significantly enhances the competitive level of snow sports, possibly due to increased international cooperation opportunities. Although fiscal expenditure promotes the development of snow sports, its effect is not significant, requiring more policy guidance and industry support. Conversely, cost expenditure significantly hinders the development of snow sports, as high costs increase operational pressure on enterprises, adversely affecting the industry (38, 39).

**Table 10:** Regression analysis of winter olympics investment impact on snow sports.

Model	FE	RE	Robustness check FE (snowsport1)
Input	0.1661*	0.1859*	0.1752*
	（1.84）	（1.89）	（1.86）
Economy	0.1593	0.1896	0.1655
	（0.18）	（0.25）	（0.20）
	0.0555*	0.0859*	0.0714*
Open	（1.75）	（1.78）	（1.81）
Finance	0.0197	0.0347	0.0254
	（1.48）	（1.59）	（1.52）
Cost	−0.0190**	−0.0398**	−0.0259**
	（−1.62）	（−1.88）	（−1.76）
C	−1.8988***	−2.5984***	−3.5587***
	（−3.87）	（−3.99）	（−4.02）
Fixed Effect	yes	yes	yes
N	318	318	318
withinR2	0.7402	0.7052	0.6539

t- or z-statistics in parentheses; *, **, and *** denote significance at the 10%, 5%, and 1% levels, respectively.

In the robustness checks, this study employed a Random Effects model (RE) and conducted a Fixed Effects regression (FE) using the transformed variable snowsport1. The regression results are shown in columns (2) and (3) of Table 10. In the RE model, the regression coefficient for Input is 0.1859, significantly positive at the 10% level; the coefficient for Economy is 0.1896, not significant; the coefficient for Open is 0.0859, significantly positive at the 10% level; the coefficient for Finance is 0.0347, not significant; the coefficient for Cost is −0.0398, significantly negative at the 5% level; the constant term coefficient is −2.5984, significantly negative at the 1% level, with a goodness-of-fit of 0.7052.In the FE (snowsport1) model, the regression coefficient for Input is 0.1752, significantly positive at the 10% level; the coefficient for Economy is 0.1655, not significant; the coefficient for Open is 0.0714, significantly positive at the 10% level; the coefficient for Finance is 0.0254, not significant; the coefficient for Cost is −0.0259, significantly negative at the 5% level; the constant term coefficient is −3.5587, significantly negative at the 1% level, with a goodness-of-fit of 0.6539. From the RE and FE (snowsport1) regression results, it is evident that the model fits well, and the core explanatory variable, Winter Olympics investment, has a significant promoting effect on the development of snow sports. The consistency with the basic regression model results indicates that the empirical conclusions are robust (40).

Marginal significance (approximately at the 10% level), as observed in variables such as Input and Open, is interpreted as offering preliminary statistical support for directional effects and trends. However, conclusions should be drawn cautiously to avoid overinterpretation, particularly given the heightened risk of Type I errors in smaller samples. Building on the robustness checks detailed above, the consistent direction and magnitude of effects across fixed effects (FE), random effects (RE), and variable-transformed models strengthen the credibility of the findings. Furthermore, the inclusion of individual effects (μi) and time effects (*σ*t) helps control for unobserved heterogeneity and mitigate potential biases from contemporaneous shocks. Potential confounders, such as regional climate conditions, macro-tourism cycles, and demographic shifts, may influence results but were not fully incorporated due to data limitations, potentially affecting causal identification (39). Future research could address these by expanding data sources and refining controls for greater precision (41, 42).

From a policy perspective, statistical significance at the 10% level is interpreted as providing directional evidence suitable for pilot programs, phased evaluations, and iterative policy calibration, rather than as a basis for one-off, large-scale resource reallocations. Consistency across RE and transformed-FE models suggests a robust positive effect of Olympic investment (43, 44). Based on these findings, it is recommended to prioritize finely tuned measures that increase openness and reduce costs, implemented incrementally with rapid evaluation cycles to minimize policy risks (45).

## 4 Discussion

The empirical findings of this study, derived from a systematically constructed evaluation framework, reveal a period of unprecedented growth in China's snow sports sector following the successful bid for the 2022 Beijing Winter Olympics. The overall development index surged by a factor of 10.92 between 2015 and 2019, with mass participation experiencing the most dramatic expansion (46). This pattern of growth, where public engagement outpaces competitive and economic dimensions, is a phenomenon observed in other contexts of mega-sporting events. The “inspirational effect” or “demonstration effect” of the Olympics, where elite athletic excellence motivates broader public participation, has been documented in previous research (47). In the Chinese context, this effect was significantly amplified and accelerated by the top-down, state-led “300 Million People in Ice and Snow Sports” campaign, creating a unique synergy between Olympic inspiration and coordinated policy implementation that may explain the exceptionally rapid growth in mass participation compared to other Winter Olympic hosts (48).

The concentric circle model developed through this research provides a robust theoretical framework for understanding the hierarchical and interconnected nature of snow sports development. This model aligns with international mega-event legacy frameworks that identify the interplay between core competition, public participation, and industrial spillover (49). However, it extends these models by incorporating the distinctive characteristics of the Chinese development pathway, which involved simultaneous investment across all three dimensions within a compressed timeframe. The empirical validation of this model through longitudinal data adds a significant contribution to the theoretical discourse on sport development, offering a replicable framework for other nations seeking to leverage mega-events for holistic sectoral growth.

The regression analysis quantifies the significant positive impact of Winter Olympics investment (*β* = 0.1661, *p* < 0.1), affirming the catalytic role of strategic mega-event investment. This finding is consistent with studies on previous Winter Games, which have shown that targeted investment can serve as a powerful driver for sport infrastructure and participation. However, the concurrent identification of cost expenditure as a significant barrier (*β* = −0.0190 to −0.0398, *p* < 0.05) highlights a critical challenge for long-term sustainability. High operational costs can erode the economic viability of resorts and facilities, particularly in regions without natural advantages, potentially threatening the longevity of the participation legacy once the immediate post-Olympic period concludes. This underscores the necessity of complementing initial investment with policies aimed at improving operational efficiency and reducing barriers to entry for both operators and participants.

While the growth trajectory is impressive, the relatively slower pace of economic development (8.82-fold increase) compared to mass and competitive growth suggests a lag in converting participation into sustainable economic value. This may reflect the initial phase of development being dominated by infrastructure investment and capacity building, with the maturation of a robust sports industry ecosystem requiring a longer timeframe. Future strategies must therefore focus on fostering innovation, developing value-added services, and enhancing the visitor experience to fully capitalize on the participation base and ensure economic sustainability in the post-Olympic era.

This study is not without limitations. The concentration on the 2015–2019 pre-Olympic period captures the build-up phase but necessitates follow-up research to assess the sustainability of these development trends beyond the immediate Olympic horizon. Furthermore, while the national-level analysis reveals macro-trends, future research incorporating more granular regional data could provide deeper insights into the varied local impacts and the role of climatic and geographic factors (50). Finally, enhancing the international comparability of the index by benchmarking against FIS standards and best practices from established winter sports nations would strengthen its global relevance.

## 5 Conclusion

This study set out to construct a scientifically-grounded evaluation system to measure the holistic development of snow sports in China, a national priority following the successful bid for the 2022 Beijing Winter Olympics. To achieve this, a mixed-methods approach was employed, whereby insights from expert interviews were analyzed via grounded theory to develop a comprehensive three-level indicator system. The application of a combined weighting method to panel data from 2015 to 2019 then enabled the computation of a unified development index, with panel regression models subsequently validating the system's utility and probing key impact mechanisms. The empirical findings reveal a period of remarkable transformation, with the overall development index surging by a factor of 10.92, catalyzed by strategic Olympic investment. This growth was most pronounced in mass participation, underscoring the success of policy-driven engagement, though significant gains were also observed in competitive and economic dimensions.

The implications of these findings are twofold. Theoretically, this research provides a validated model that contributes to the understanding of sport development pathways, particularly in the context of mega-events. Practically, it offers policymakers and stakeholders an evidence-based framework for strategic planning and resource allocation in the post-Olympic era, highlighting the proven efficacy of targeted investment while also sounding a note of caution regarding the inhibitory effect of high costs on long-term sustainability. Consequently, future strategies must evolve from building infrastructure to optimizing operational efficiency and enhancing the consumer experience to foster a resilient and self-sustaining market.

It is important to acknowledge the limitations of this work, which in turn illuminate pathways for future research. The concentration on the pre-Olympic build-up phase necessitates longitudinal studies to track the sustainability of these trends beyond the immediate event horizon. Furthermore, the national-level scope of the analysis, while revealing macro-trends, invites more granular investigation into the disparate regional impacts shaped by local factors. Extending this line of inquiry through international benchmarking against global standards would further enhance the model's applicability. Thus, future research should prioritize these longitudinal, regional, and comparative dimensions to build upon the foundation established here.

## Data Availability

The original contributions presented in the study are included in the article/Supplementary Material, further inquiries can be directed to the corresponding authors.

## References

[1] Bu P Chen G Kong J. Beijing Winter Olympics: the enlightenment of ice and snow culture and the promotion strategy of sports tourism industry—summary of the academic workshop “Beijing winter olympics and the development of Chinese ice and snow culture” in sports and science. Sports Sci. (2021) 42(06):1–5. 10.13598/j.issn1004-4590.2021.06.001

[2] China GN. General administration of sport on the issuance of the “14th five-year plan for sports development” notice. Gen Admin Sport. (2021). http://www.gov.c/henge/zhengceku/2021-10/26/content_5644891.htm

[3] Guo F Yi J Ren H. Mobilization governance: the development strategy of China’s mass ice and snow projects in the post-winter olympics era. J Guangzhou Inst Phys Educ. (2023) 43(5):1–10. 10.13830/j.cnki.cn44-1129/g8.2023.05.001

[4] Xi YY. Jinping attends the opening ceremony of the 9th Asian winter games and announces the opening of the event. Xinhua News Agency. (2025). http://www.gov.c/yaowen/tupian/202502/content_7002735.htm

[5] He L Ru X. Research on the “China plan” for the implementation and evaluation of Beijing 2022 Olympic education. J Cap Phys Educ Inst. (2023) 35(05):530–41. 10.14036/j.cnki.cn11-4513.2023.05.008

[6] Jin Z. The connotation, times characteristics and inheritance path of Beijing winter Olympic spirit. J Beijing Sport University. (2023) 46(03):99–109. 10.19582/j.cnki.11-3785/g8.2023.03.009

[7] Sun B Yan J Ye F Zhang X Zhang H Zhang B A study on the sustainable management of Beijing winter Olympic heritage in the post-winter Olympic era. J Shandong Phys Educ Inst. (2024) 40(6):1–7. 10.14104/j.cnki.1006-2076.2024.06.01

[8] Zou X. Study on post-competition utilization mode of Olympic venues in France. J Nanjing University Phys Edu (Social Science Edition). (2019) 5(1). 10.3969/j.issn.1008-1909.2019.01.005

[9] Zhang R. Construction of coordinated ecological civilization of ice and snow tourism in the new era: logical approach and promotion strategy. Phys Educ Res. (2024) 38(2):52–62. 10.15877/j.cnki.nsic.20240429.002

[10] Zhao J Li Z. Research on the development of ice and snow sports in China under the background of sports power construction. J Chengdu Physical Education University. (2024) 50(4):88–94. 10.15942/j.jcsu.2024.04.011

[11] Yi N Zhang W Ye S Qiu Z. The development and evolution of winter Olympic events and the selection of Chinese Olympic events. J Beijing Sport University. (2018) 41(05):1–8. 10.19582/j.cnki.11-3785/g8.2018.05.001

[12] Qiu Z Yin Y Ye M Meng Q Qiu S. Summer training measures to improve the special ability of Chinese winter Olympic athletes. J Beijing Sport University. (2021) 44(3):15. 10.19582/j.cnki.11-3785/g8.2021.03.001

[13] Cheng Y Fan Y. The theory and logic of supply and demand balance of sports industry under the new development pattern, the attribution of difficulties and countermeasures. J Wuhan Phys Educ Inst. (2023) 57(6):54–61. 10.15930/j.cnki.wtxb.2023.06.007

[14] Liu Y. Two general modes of attribution therapy. J Health Psychol. (1998) 42(03):335–7. 10.13342/j.cnki.cjhp.1998.03.063

[15] Cui Y. An experimental study on the implementation of attribution training in calisthenics elective courses in colleges and universities. J Shandong Phys Educ Inst. (2010) 26(08):90–6. 10.14104/j.cnki.1006-2076.2010.08.019

[16] Zhao D Chen L Pan L. Research on industry correlation of sports investment in China. J Wuhan Phys Educ Inst. (2010) 44(10):4. 10.15930/j.cnki.wtxb.2010.10.011

[17] Liu M. A study on the relationship between high school students’ self-esteem level and their academic and interpersonal success and failure attributions. Psychol Sci. (1998) 23(03):281–2. 10.16719/j.cnki.1671-6981.1998.03.029

[18] Chen Y Bao P Zhou B Liu H Shi X Chen L Research on construction of evaluation index system for public sports venue operators. J Cap Phys Educ Inst. (2024) 36(04):385–97. 10.14036/j.cnki.cn11-4513.2024.04.004

[19] Deng R Zhou L. Analysis of consumer behavior path of ice and snow sports in south China—based on the perspective of method goal chain theory. J Wuhan Phys Educ Inst. (2022) 56(3):54–60. 10.15930/j.cnki.wtxb.2022.03.007

[20] Hu J Zhang W Chen S. Attribution of attention loss in fragmented learning of college students: a qualitative analysis based on grounded theory. Res Audio Vis Educ. (2019) 40(12):36–43. 10.13811/j.cnki.eer.2019.12.005

[21] Guan J Wang F Zhou Y. Influencing factors and empirical analysis of public service collaborative governance demands of ice and snow sports. Chinese Sports Sci Technol. (2023) 59(10):88–96. 10.16470/j.csst.2023038

[22] Zhao D Zhou L. The process model construction of ice and snow winter camp to improve adolescents’ subjective well-being: an exploratory analysis based on grounded theory. Phys Educ J. (2023) 30(6)):97–103. 10.16237/j.cnki.cn44-1404/g8.20230928.004

[23] Fan C Ye D Li H. Research on the formation mechanism of consumer coping behavior in product injury crisis: rooted analysis based on PADM theory. Manage Rev. (2019) 31(08):230–9. 10.14120/j.cnki.cn11-5057/f.2019.08.020

[24] Yuan J Ma L. How to protect sports intangible cultural heritage effectively?—grounded theory and qualitative comparative analysis based on 30 cases across the country. Sports Sci. (2024) 26(10):16–36. 10.16469/J.css.2024KX032

[25] Zhang X Fan X. Identity and reflection balance: the theoretical logic of national traditional sports promoting the generation of cultural self-confidence. J Shanghai Sport University. (2024) 48(9):56–70. chi. 10.16099/j.sus.2023.11.13.0003

[26] Chen X. Exploration of the application of grounded theory in Chinese education research. Peking University Educ Rev. (2015) 13(01):2–15 + 188. 10.19355/j.cnki.1671-9468.2015.01.002

[27] Zeng W Yang X. Actors, translators and networks: xinhua news production for the Tokyo olympics and its international cooperation. J Chengdu Phys Educ University. (2024) 50(04):64–71. 10.15942/j.jcsu.2024.04.008

[28] Wang Z Zheng Y. The concept and type of semantic relation in information organization. Library Work Res. (2013) 18(11):13–9. 10.16384/j.cnki.lwas.2013.11.007

[29] Liang J Wang L Feng G. Governance performance of national fitness in urban communities under the guidance of health in the new era: evaluation system construction and empirical case. J Shandong Phys Educ Inst. (2024) 40(4):18–28. 10.14104/j.cnki.1006-2076.2024.04.003

[30] Xu H Zhang X Li X Chen D Zhang X Wang X Construction of comprehensive evaluation method of concise adolescent health index. School Health in China. (2023) 44(05):706–710 + 714. 10.16835/j.cnki.1000-9817.2023.05.015

[31] Zhu K Ma S Wang Y Wang Y. Winter olympics to promote employment: international mirror and China’s strategy. J Chengdu Phys Educ University. (2024) 50(1):53–60. 10.15942/j.jcsu.2024.01.007

[32] Jiang Y Wang S Shi P Jiang L. Research on sustainable use of venues heritage of Beijing winter Olympic games with digital empowerment. Tianjin Inst Phys Educ. (2025) 43(1):78–85. 10.13297/j.cnki.issn1005-0000.2025.01.010

[33] Jiaming L Junlin L. Research on the mechanism of bilateral olympic cooperation to promote the coordinated development of Beijing, Tianjin and Hebei-based on the perspective of industrial upgrading. J Cap Phys Educ Inst. (2024) 36(4):398–406. 10.14036/j.cnki.cn11-4513.2024.04.005

[34] Zhang R Jin L Wang Z. Evaluation and influencing factors of the integrated development of ski industry and tourism industry in China. J Cap Phys Educ Inst. (2024) 36(01):1–11 + 42. 10.14036/j.cnki.cn11-4513.2024.01.001

[35] Liu Z Zhang S Li L Hu B Liu R Zhao Z Research on the construction and prediction of China’s national fitness development Index system under social reform. Front Public Health. (2022) 10:878515. 10.3389/fpubh.2022.878515 35651855 PMC9149160

[36] Cai M Austria RS Gunaban MGB. Application model system of ice and snow sports intelligent tourism on account of big data. Int J Front Eng Technol. (2022) 4(7):74–9. 10.25236/IJFET.2022.040715

[37] Spörri J Bonell Monsonís O Balsiger P Bahr R Dios C Engebretsen L International ski and snowboard federation (FIS) consensus statement on training and testing in competitive alpine and freestyle skiers and snowboarders. BMJ Open Sport Exerc Med. (2025) 11(3):e002623. 10.1136/bmjsem-2025-002623 PMC12410619 40919404

[38] Liu P Zeng J. Research on the development path of ice and snow sports entering campus under the background of the Beijing winter olympics games. Int J New Dev Educ. (2023) 5(25):5–10. 10.25236/IJNDE.2023.052502

[39] Ye H. Research on the strategy of ice and snow sports from the perspective of scientific and technological development. Front Sport Research. (2022) 4(1):12–6. 10.25236/FSR.2022.040103

[40] Deering KN Chettiar J Chan K Taylor M Montaner JS Shannon K. Sex work and the public health impacts of the 2010 Olympic games. Sex Transm Infect. (2012) 88(4):301–3. 10.1136/sextrans-2011-050235 22436199 PMC3378328

[41] Laursen L. Paris Olympics host a new event: algorithmic video surveillance: security olympics spin-offs are coming for you, and you, and you. IEEE Spectr. (2024) 61(01):34–7. 10.1109/MSPEC.2024.10380465

[42] Brownlie L. Aerodynamic drag reduction in winter sports: the quest for “free speed”. Proc Inst Mech Eng Part P J Sports Eng Technol. (2021) 235(4):365–404. chi. 10.1177/1754337120921091

[43] Kietlinski R. ‘A strong, sustainable legacy:’ the environment and Japan’s winter olympics. Int J History Sport. (2021) 38(13–14):1476–93. 10.1080/09523367.2021.1958784

[44] Scott D Knowles NLB Ma S Rutty M Steiger R. Climate change and the future of the Olympic winter games: athlete and coach perspectives. Curr Issues Tourism. (2023) 26(3):480–95. 10.1080/13683500.2021.2023480

[45] Ruedl G Schnitzer M Kirschner W Spiegel R Platzgummer H Kopp M Sports injuries and illnesses during the 2015 winter European youth Olympic festival. Br J Sports Med. (2016) 50(10):631–6. 10.1136/bjsports-2015-095665 26884224

[46] Ding Z. An analysis of snow sports promotion in China through different social media platforms. Open J Bus Manag. (2025) 13(01):489–501. 10.4236/ojbm.2025.131027

[47] Zhao W Liu H Wang D Xu G Xu C Li J The global research status and trends in ice and snow sports injuries from 1995 to 2022: a bibliometric and visualized analysis. Int J Environ Res Public Health. (2023) 20(04):2880–2880. 10.3390/ijerph20042880 36833576 PMC9957478

[48] Ge Y Li T. Research on the brand building of jilin province ice and snow sports equipment industry under the background of all-for-one tourism. Tourism Manag Technol Econ. (2022) 5(03):85–94. 10.23977/tmte.2022.050314

[49] Tang C Zeng R Yang Y Xu S Wang X. High-quality development paths of ice-snow tourism in China from the perspective of the winter olympics. J Resour Ecol. (2022) 13(04):552–63. 10.5814/j.issn.1674-764x.2022.04.002

[50] Chen X Xu S Tang C Fu Z Xu X. Evaluation and promotion model of tourist satisfaction in ice and snow tourism destinations. J Resour Ecol. (2022) 13(04):635–45. 10.5814/j.issn.1674-764x.2022.04.009

