# Supplementary material for: Construction and empirical study of China's snow sports evaluation Index system
Source: Front Sports Act Living. 2025 Nov 7;7:1619136. doi: 10.3389/fspor.2025.1619136 (PMC12634621; doi:10.3389/fspor.2025.1619136)
Supplement: Supplementary file 1 [file Datasheet1.docx]

**Appendix 1: Web Scraping Code for Acquiring Relevant Data**

*# Web Scraping Code*

*import os*

*import json*

*import csv*

*import requests*

*from lxml import etree*

*if 'data.csv ' not in os.listdir():*

*with open('data.csv ', 'w ', newline= ' ', encoding='utf-8_sig ') as f: csv_f = csv.writer(f)*

*csv_f.writerow(['Serial Number', 'Company Name', 'Industry', 'Region', 'Registered Capital', 'Establishment Date', 'Registered Address'] + ['15 Number of Employees', '16 Employee Count', '17 Employee Count', '18 Employee Count', '19 Employee Count', '20 Employee Count', '21 Employee Count'])*

*old = []*

*xuhao = 1 else:*

*with open('data.csv ', 'r ', newline= ' ', encoding='utf-8_sig ') as f: csv_f = csv.reader(f)*

*old = [i[-1] for i in csv_f] xuhao = len(old)*

*while True:*

*eles = driver.find_elements_by_xpath("//a[@class='title '] ") for ele in eles:*

*href = ele.get_attribute('href') if href in old:*

*continue ele.click()*

*driver.switch_to_window(driver.window_handles[1])*

*status = 0*

*for i in range(8): time.sleep(1) try:*

*driver.find_element_by_xpath("//*[text()='Trend Chart> '] ").click() status = 1*

*break except:*

*pass*

*if status == 1:*

*# Monitoring data appears*

*keyNo = driver.current_url.split('/')[-1].split('. ')[0]*

*filename = keyNo + '.txt ' while True:*

*if os.path.exists('hook Program' + os.sep + filename): break*

*# Read data*

*with open('hook Program' + os.sep + filename, encoding='utf-8 ') as f: canbaoshuju = json.loads(f.read())*

*else:*

*canbaoshuju = [] try:*

*yuangong15 = [i for i in canbaoshuju['Result '] if i['ReportYear '] == '2015 '][0]['InsuredCount '] except:*

*yuangong15 = ' ' try:*

*yuangong16 = [i for i in canbaoshuju['Result '] if i['ReportYear '] == '2016 '][0]['InsuredCount '] except:*

*yuangong16 = ' ' try:*

*yuangong17 = [i for i in canbaoshuju['Result '] if i['ReportYear '] == '2017 '][0]['InsuredCount ']*

*except:*

*yuangong17 = ' ' try:*

*yuangong18 = [i for i in canbaoshuju['Result '] if i['ReportYear '] == '2018 '][0]['InsuredCount '] except:*

*yuangong18 = ' ' try:*

*yuangong19 = [i for i in canbaoshuju['Result '] if i['ReportYear '] == '2019 '][0]['InsuredCount '] except:*

*yuangong19 = ' ' try:*

*yuangong20 = [i for i in canbaoshuju['Result '] if i['ReportYear '] == '2020 '][0]['InsuredCount '] except:*

*yuangong20 = ' ' try:*

*yuangong21 = [i for i in canbaoshuju['Result '] if i['ReportYear '] == '2021 '][0]['InsuredCount '] except:*

*yuangong21 = ' '*

*html = etree.HTML(driver.page_source)*

*ziduanshuju = [xuhao, html.xpath("//h1 ")[0].xpath("string() ").strip()] # Convenience fields*

*for ziduan in ['Industry', 'Region', 'Registered Capital', 'Date of Establishment', 'Registered Address']: try:*

*shuju = html.xpath("//*[@id='cominfo ']//*[contains(text(), '%s ')]/following- sibling::*[1] " % ziduan)[0].xpath( "string() ").strip()*

*except:*

*shuju = ' '*

*ziduanshuju.append(shuju)*

*ziduanshuju += [yuangong15, yuangong16, yuangong17, yuangong18, yuangong19, yuangong20, yuangong21, driver.current_url]*

*with open('data.csv ', 'a ', newline= ' ', encoding='utf-8_sig ') as f: csv_f = csv.writer(f)*

*csv_f.writerow(ziduanshuju)*

*xuhao += 1*

*time.sleep(4) driver.close()*

*driver.switch_to_window(driver.window_handles[0])*

*try:*

*driver.find_element_by_xpath("//*[text()='> '] ").click() time.sleep(4)*

*except: break*

**Appendix 2: Expert Consultation Questionnaire on Data Objectivity and Reasonableness**

Dear Expert：

Hello, our writing team is conducting research for a paper. To verify the objectivity and reasonableness of the relevant data, and based on your achievements and status in this field, we hope you can take some valuable time from your busy schedule to fill out this questionnaire. The questionnaire is divided into two parts: the first part is your basic information, and the second part consists of 54 indicators within our research system, using a five-point Likert scale. Please assess their objectivity and reasonableness based on your professional knowledge and experience. The information you provide will only be used for this project and your personal information will not be disclosed. There are no right or wrong answers; please respond objectively based on your knowledge and judgment. If there are any shortcomings, we hope you can provide guidance. Thank you again for your strong support and assistance!

Writing Team

I. Your Basic Information

(1) Name:

(2) Gender: A. Male B. Female

(3) Identity: A. Government Staff B. Enterprise Manager C. Academic Scholar in Related Fields

(4) Affiliation:

II. Questionnaire on Winter Sports Development Indicators

(1) Please read each data item carefully before responding.

(2) Please evaluate each data set and rate its reliability (5 > 4 > 3 > 2 > 1), where 5 represents "reasonable," 4 "somewhat reasonable," 3 "uncertain," 2 "somewhat unreasonable," and 1 "unreasonable."

(3) If you find any year's data unreasonable, please provide the data range you consider appropriate in the remarks column and state the relevant reasons.

(4) The third item in the questionnaire, "Winter Sports World Cup," is a cross-year event. To ensure consistency in the header, "2015" shows data for the "2014-2015 season," and so on.

(5) All data below are domestic. For simplicity, the word "domestic" is omitted from all item names.

**Table: 2015-2019 Questionnaire on Winter Sports Development Indicators**

| Tertiary Indicators of the Winter Sports Development Index System | 2015 | 2016 | 2017 | 2018 | 2019 | Do you think this set of data is objective and reasonable? (5 > 4 > 3 > 2 > 1)  1 2 3 4 5 | Remark column |
| --- | --- | --- | --- | --- | --- | --- | --- |
| Number of Snow Sports Medals at the Winter Olympics | \ | \ | \ | 4 | \ |  |  |
| Number of Snow Sports Medals at the World Championships | 3 | \ | 3 | \ | 2 |  |  |
| Number of Snow Sports Medals at the World Cup | 10 | 10 | 9 | 8 | 8 |  |  |
| Number of Ski Events | 96 | 112 | 176 | 251 | 279 |  |  |
| Professional Competitions | 36 | 43 | 51 | 59 | 68 |  |  |
| Public Competitions | 53 | 66 | 117 | 128 | 139 |  |  |
| Number of Event Parameter Units | 77 | 88 | 96 | 106 | 148 |  |  |
| Number of National Team Athletes | 526 | 605 | 778 | 869 | 979 |  |  |
| Number of Competitive Service Personnel | 204 | 242 | 302 | 338 | 382 |  |  |
| Number of National Snow Sports Training Bases Accredited by the Winter Sports Center | 1 | 1 | 1 | 1 | 7 |  |  |
| Number of Research Projects Related to Competitive Snow Sports | 8 | 15 | 18 | 22 | 26 |  |  |
| Number of FIS-Certified Slopes in Chinese Ski Resorts | 18 | 26 | 26 | 29 | 29 |  |  |
| Number of Ski Resort Visits | 1250 | 1510 | 1750 | 1970 | 2090 |  |  |
| Number of Ski Simulator Visits | 2.1 | 6.72 | 24.15 | 58 | 78 |  |  |
| Number of Dry Ski Visits | 3.9 | 4.82 | 10.65 | 22.3 | 34.23 |  |  |
| Number of Skiers | 960 | 1133 | 1210 | 1320 | 1305 |  |  |
| Annual Average Skiing Frequency | 1.3 | 1.3 | 1.4 | 1.5 | 1.6 |  |  |
| Number of Ski Social Sports Instructors | 356 | 458 | 569 | 688 | 798 |  |  |
| Number of Ski Enterprise Employees Paying Social Security | —— | 64207 | 68641 | 120932 | 74909 |  |  |
| Number of Domestic Ski Resorts | 568 | 646 | 703 | 742 | 770 |  |  |
| Number of Ski Resorts with Aerial Cableways | 109 | 125 | 145 | 149 | 155 |  |  |
| Total Number of Aerial Cableways in Ski Resorts | 179 | 199 | 236 | 250 | 261 |  |  |
| Number of Ski Resorts with Detachable Cableways | 10 | 12 | 18 | 19 | 22 |  |  |
| Total Number of Detachable Cableways | 26 | 35 | 48 | 54 | 60 |  |  |
| Number of Imported Detachable Aerial Cableways | 24 | 29 | 33 | 36 | 40 |  |  |
| Number of Domestic Detachable Aerial Cableways | 2 | 6 | 15 | 18 | 20 |  |  |
| Number of New Magic Carpets in Ski Resorts | 164 | 232 | 226 | 120 | 140 |  |  |
| Length of New Magic Carpets in Ski Resorts | 21.8 | 36.3 | 28.7 | 19 | 21 |  |  |
| Total Number of Operating Magic Carpets in Ski Resorts | 618 | 850 | 1076 | 1196 | 1336 |  |  |
| Total Length of Operating Magic Carpets in Ski Resorts | 92.1 | 128.3 | 157 | 176 | 197 |  |  |
| Number of New Imported Snow Groomers in Ski Resorts | 61 | 56 | 48 | 36 | 61 |  |  |
| Number of New Domestic Snow Groomers in Ski Resorts | 10 | 20 | 22 | 20 | 27 |  |  |
| Number of New Imported Second-Hand Snow Groomers in Ski Resorts | 0 | 4 | 5 | 0 | 0 |  |  |
| Number of New Imported Snowmaking Machines in Ski Resorts | 650 | 1080 | 1220 | 510 | 682 |  |  |
| Number of New Domestic Snowmaking Machines in Ski Resorts | 50 | 100 | 200 | 300 | 467 |  |  |
| Number of Indoor Ski Resorts | 14 | 18 | 21 | 26 | 31 |  |  |
| Number of Dry Ski Resorts | 14 | 18 | 21 | 32 | 45 |  |  |
| Area of Dry Ski Facilities | 6.2 | 6.43 | 12.32 | 15.24 | 16.78 |  |  |
| Number of Ski Simulators | 6 | 21 | 69 | 145 | 400 |  |  |
| Twin Tip Binding Imports (Customs Data, Unit: Tons) | 106.14 | 113.43 | 137.45 | 92 | 119 |  |  |
| Number of Ski Enterprises | 2817 | 2879 | 3025 | 2972 | 3039 |  |  |
| Number of Snow Sports Clubs | 396 | 481 | 872 | 1065 | 1172 |  |  |
| Number of Ice and Snow Sports Specialty Schools | 0 | 227 | 440 | 713 | 1036 |  |  |
| Number of Universities Offering Ice and Snow-Related Majors | 7 | 12 | 19 | 22 | 26 |  |  |
| Baidu Search Index | 379241 | 429468 | 446227 | 476585 | 478415 |  |  |
| Comprehensive Communication Index (WCI) of Leading Ski WeChat Public Accounts | 1269 | 1429 | 1658 | 1856 | 2057 |  |  |
| Registered Capital of Ski Enterprises | 755 | 862 | 1030 | 1193 | 1340 |  |  |
| Per Capita Consumption in Domestic Ski Tourism | 4259 | 4899 | 5299 | 5896 | 6298 |  |  |
| Scale of the Equipment Market | 48 | 68 | 84.7 | 105.6 | 117.5 |  |  |
| Scale of the Training Market | 50.1 | 59.1 | 68.2 | 68.9 | 69.1 |  |  |
| Scale of the Event Market | 36.2 | 58.1 | 77.4 | 98 | 110 |  |  |
| Price-to-Earnings Ratio of Listed Snow Sports Companies | 79.51 | 82.55 | 84.75 | 91.62 | 95.71 |  |  |
| Market Value of Listed Snow Sports Companies | 712.59 | 742.51 | 760.45 | 790.65 | 820.62 |  |  |
| Stock Price of Listed Snow Sports Companies | 10.85 | 11.59 | 13.52 | 13.95 | 14.25 |  |  |

Filling time:
